# Association Between Gambling Motives, Violence and Early Maladaptive Schemas in Women with Gambling Disorder

**DOI:** 10.1007/s10899-024-10285-8

**Published:** 2024-03-01

**Authors:** A. Estévez, P. Jauregui, J. Momeñe, L. Macía

**Affiliations:** University of Deusto, Avda. de las Facultades, 24, 48007 Bilbao, Spain

**Keywords:** Gambling motives, Gambling disorder, Violence, Early maladaptive schemas, Women

## Abstract

Analyzing why being a victim of violence has led women to have problems with gambling is a field yet to be explored. Thus, the objectives of the present study were (I) analyze the relationship between gambling motives, received psychological violence, and early maladaptive schemas in women; (II) study differences in the study variables in women with and without gambling disorder (GD); (III) analyze the predictive role of violence and schemas in gambling motives; and (IV) analyze the mediating role of schemas in the relationship between violence and gambling motives. The sample comprised 61 women with GD (*M* = 48.43, *SD* = 12.78) and 342 women without GD (*M* = 26.91, *SD* = 11.47). The results of the present study revealed positive correlations between gambling motives, psychological violence received and early maladaptive schemas. In addition, women with GD scored higher on the study variables. It was also found that early maladaptive schemas based on subjugation and defectiveness may be a vulnerability factor for engaging in gambling to cope with the negative emotions produced by gender violence. From a clinical perspective, knowing the risk factors related to gambling motives in women is crucial to developing effective prevention and intervention programs.

## Introduction

Women's participation in gambling behaviors has increased in recent years due to sociocultural, environmental, and commercial factors (McCarthy et al., 2023). Gambling is increasingly normalized among women because gambling products are being designed to appeal to female audiences. In turn, it is easy to consume and can be accessed from home and anonymously. It is easy to establish a routine in gambling behavior and gamble automatically (Thomas et al., 2022). Thus, problem gambling among women is becoming increasingly prevalent, and the damage caused in different areas of women's lives is becoming increasingly severe (McCarthy et al., 2018). Because of this, the importance of conducting an in-depth analysis of the motivational factors that drive women to gamble has been studied (Leonek-Kuleta, 2022) to develop effective prevention and intervention programs (Esparza-Reig et al., 2022). In this regard, motives for gambling are shown to be associated with engaging in gambling and are, therefore, crucial for understanding the etiology of problem gambling (Sundqvist et al., 2016). Thus, there are specific motivations or reasons that lead women to engage in gambling activities (Mathieu et al., 2018; Rash et al., 2017).

In this line, previous studies have shown that women gamble mainly to escape from their daily lives, for economic reasons, primarily to change their personal circumstances, and to socialize or as a leisure activity (McCarthy et al., 2023). For her part, Leonek-Kuleta (2022) pointed out that the motivation to engage in gambling behaviors among women addicted to gambling is related to sensations, escape, and socialization. Similarly, Kim et al. (2016) mentioned that women are motivated to gamble to escape from the loneliness and isolation they experience as they age. Therefore, preliminary results indicate the centrality of relational aspects in women's excessive involvement in gambling (Odoardi & Albasi, 2013). Another recent study has mentioned differences in gambling motives among women as a function of women's age. While searching for positive feelings and socialization were the most frequent motives among young female gamblers, motives of escape and support for noble causes were more frequent among housewives and older women (Hagfors et al., 2022). Also, it has been mentioned that middle-aged women who gamble for money or entertainment are the high-risk group for developing a gambling addiction (Hing et al., 2016). It should be noted that, although men also gamble to win money as in the case of women, previous studies seem to highlight a greater tendency for men to engage in gambling behaviors due to sensation seeking, impulsivity and excitement (Corral et al., 2005). In contrast, women, as mentioned above, gamble to win money, but gambling is predominantly initiated to cope with personal problems, cope with dysphoric moods (e.g., depression, anxiety, anger, etc.), as well as to relate to other people (Hing & Breen, 2001).

There is empirical evidence that women's gambling could be a response to intimate partner violence. The violence received is a severe traumatic experience for women that would drive them to play as a way to physically and psychologically escape from reality. In addition, it has been found that playgrounds are more attractive to women who suffer gender-based violence (Dowling et al., 2016; Hing et al., 2023; Zhai et al., 2020). The findings obtained so far indicate that both issues worsen over time. That is, gender-based violence predicts women’s gambling, and problem gambling, in turn, exacerbates violence and coercive control by the partner. Likewise, older female gamblers have been found to experience more gender-based violence. This could be related to traditional views of marriage and rigid gender expectations that favor silence about violence, reluctance to disclose experiences of violence, and beliefs related to deserving abuse (Hing et al., 2022). The most frequently received violence is psychological violence, although there are also high rates of physical violence (Suomi et al., 2019). Therefore, the need to reduce problem gambling to decrease gambling-related gender violence has been observed (Hing et al., 2023). However, many authors criticize the lack of research exploring the factors associated with the coexistence of problem gambling and gender-based violence (Dowling et al., 2021; O'Mullan et al., 2022; Suomi et al., 2019). Understanding the factors involved in this relationship could lead to better identification and intervention (Roberts et al., 2018).

Recently, the vital role played by early maladaptive schemas in developing various behavioral addictions has begun to be analyzed. Young et al. (2013) pointed out that each problematic or dysfunctional behavior is sustained by specific dysfunctional schemas. He defined them as beliefs that include lived experiences, memories, emotions, feelings, and bodily sensations about oneself and others that reorganize information and influence how one feels, thinks, and behaves with others throughout life. Schemas are consolidated at very early ages and remain relatively stable throughout life, acting as frozen learning. Studies agree that the "disconnection and rejection" schema is the schema most strongly related to all behavioral addictions, followed by "impaired boundaries" and "impaired autonomy" (Sakulsriprasert et al., 2023; Viera et al., 2023). Specifically, gambling addiction has recently been linked to "autonomy impairment" (Aloi et al., 2020). However, little is known about their impact on the motivation to engage in gambling behaviors, especially in female gamblers. Delineating early maladaptive schemas associated with women’s motivation to gamble could improve therapeutic and gambling prevention interventions (Cudo et al., 2020).

Previous literature points to the relationship between gender-based violence and women’s involvement in gambling behaviors. However, few studies analyze why being a victim of violence has led women to have gambling problems. Although early maladaptive schemas seem to play an important role in problem gambling, their possible influence on women's gambling behavior remains to be explored. Consequently, the objectives of the present study were: (I) to analyze the correlations between the study variables; (II) to analyze the mean differences in gambling motives, received psychological violence and early maladaptive schemas between the clinical and control samples; (III) to analyze the predictive role of received psychological violence and schemas in gambling motives; and (IV) to analyze the mediating role of schemas in the relationship between violence and gambling motives (Table 1).

**Table 1:** Sociodemographic data

	Clinical sample (*n* = 61)	Control sample (*n* = 342)
*Level of education*		
No education	3.3%	0
Primary education	32.8%	0.3%
Secondary education	16.4%	17.1%
Vocational training	29.5%	8.8%
University education	4.2%	73.7%
*Employment status*		
Working	47.5%	23%
Unemployed	23%	2.7%
Students	1.6%	49.3%
Retired	1.2%	13.1%
Studies and work	21.8%	1.6%
Pensioners	1.2%	0.5%
Another status	9.8%	0.9%
*Marital status*		
Single	42.6%	48%
Married	31.1%	8.2%
Separated	18%	3.2%
Widowed	4.9%	1.2%
Other marital status	3.3%	35.7%

## Method

### Participants

The sample consisted of 61 women with gambling disorder (GD) who constituted the clinical sample (age: *M* = 48.43, *SD* = 12.78), and 342 women without GD who made up the control sample (age: *M* = 26.91, *SD* = 11.47), recruited by convenience sampling. In the case of the clinical sample, the level of education was as follows: no education (3.3%), primary education (32.8%), secondary education (16.4%), vocational training (29.5%), and university education (4.2%). Regarding employment status, 47.5% reported working, 23% were unemployed, 1.6% were students, 1.2% were retired, 21.8% combined studies and work, 1.2% were pensioners, and 9.8% reported another status. Regarding marital status, 42.6% were single, 31.1% were married, 18% were separated, 4.9% were widowed, and 3.3% had other marital statuses. In the case of the control sample, the level of education was as follows: primary education (0.3%), secondary education (17.1%), vocational training (8.8%), university education (73.7%). Regarding employment status, 23% reported being employed, 2.7% were unemployed, 49.3% were students, 13.1% were retired, 1.6% were simultaneously studying and working, 0.5% were pensioners, and 0.9% reported another status. Regarding marital status, 48% were single, 8.2% were married, 2.6% were in a domestic partnership, 3.2% were separated, 1.2% were widowed, and 35.7% had other marital statuses.

In the case of the sample with GD, the inclusion criterion was to attend treatment at a GD center and to score as a gambler on the South Oaks Gambling Screen (SOGS) (Lesieur & Blume, 1987). In the case of the sample without GD, the exclusion criterion was not scoring as a gambler on the SOGS. In all cases, being of legal age was a requirement to participate in the study.

### Instruments

#### Gambling Motives

The *Gambling Motives Questionnaire* (GMQ; Stewart & Zack, 2008) was translated into Spanish by Jauregui et al. (2018). This scale comprises 15 items that categorize gambling problems into three subscales of gambling motives: Social motives (e.g., “because it’s what my friends do when we meet”); Enhancement motives (e.g., “obtaining an intense feeling”) and Coping motives (e.g., “because it helps you when you feel overwhelmed or depressed”). Every item is an adaptation of the *Drinking Motive Questionnaire* (Cooper, 1994), scored on a 4-point Likert scale ranging from 1 (*never/almost never*) to 4 (*almost always*). All the subscales had good internal consistency (*α* > 0.80). In this study, Cronbach's alpha ranged from 0.84 to 0.87.

#### Violence

*Inventory of Psychological Abuse in Couple Relationships* (IAPRP; Calvete et al., 2005). The inventory consists of 17 items. Respondents report the frequency with which each type of abuse occurred during the past year: 1 (*once in the past year*), 2 (*twice in the past year*), 3 (*3–5 times in the past year*), 4 (*6–10 times in the past year*), 5 (*11–20 times in the past year*), 6 (*more than 20 times in the past year*), 7 (*never in the past year, but before*), and 0 (*never occurred*). The inventory provides annual prevalence, chronicity, and annual frequency indices. Cronbach's alpha was 0.99 (Calvete et al., 2005). Cronbach's alpha in the present study was 0.92.

#### Early Maladaptive Schemas

*Schema Questionnaire—Short Form* (Young & Brown, 1994). This questionnaire evaluates the 15 schemas proposed by Young classified into four domains. This study evaluated the following schemas: *Domain of Disconnection/Rejection:* (1) Emotional Deprivation: it includes the expectation that one’s desire for a normal degree of emotional support will not be adequately met by others; (2) Abandonment: The perceived instability or unreliability of those available for support and connection; (3) Abuse: The expectation that others will hurt, abuse, humiliate, cheat, lie, manipulate, or take advantage of one. It usually involves the perception that the harm is intentional or the result of unjustified and extreme neglect; (4) Defectiveness: The feeling that one is defective, bad, unwanted, inferior, or invalid in important respects or that one would be unlovable to significant others if exposed; (5) Social Isolation: The feeling that one is isolated from the rest of the world. *Domain of Impaired Autonomy:* (6) Failure: The belief that one has failed, will inevitably fail, or is fundamentally inadequate compared to one's peers in terms of achievement; (7) Dependence: The belief that one is unable to handle everyday responsibilities competently without considerable help from others; (8) Enmeshment: Excessive emotional involvement and closeness with one or more significant others at the expense of full individuation or normal social development. *Domain of Other-Directedness:* (9) Subjugation: Excessive surrendering of control to others because one feels coerced, usually to avoid anger, retaliation, or abandonment; (10) Self-sacrifice: Excessive focus on voluntarily meeting the needs of others in daily situations at the expense of one's own gratification. *Domain of Overvigilance and Emotional Inhibition:* (11) Emotional Inhibition: Excessive inhibition of spontaneous action, feeling, or communication, usually to avoid disapproval by others, feelings of shame, or losing control of one's impulses. The higher the score, the higher the early dysfunctional schema. The Spanish version of the SQ-SF presents good psychometric properties (Calvete et al., 2005). The short form used in this study was developed using items from the long form of the SQ that were translated to Spanish by Cid and Torrubia (2002) in collaboration with Young. The Spanish version of the SQ-SF has shown good reliability, obtaining a Cronbach's alpha coefficient for the total scale of 0.97 (Iruarrizaga et al., 2019). In this study, the Cronbach's alpha coefficients were: Enmeshment (*α* = 0.78), Abandonment (*α* = 0.87), Abuse (*α* = 0.85), Emotional Deprivation (*α* = 0.89), Emotional Inhibition (*α* = 0.84), Social Isolation (*α* = 0.78), Subjugation (*α* = 0.83), Self-Sacrifice (*α* = 0.86), Failure (*α* = 0.87), Defectiveness (*α* = 0.80), and Dependence (*α* = 0.78).

### Procedure

Clinical participants with a GD diagnosis were out-patients recruited through GD treatment associations belonging to the FEJAR (Spanish Federation of Rehabilitated Gamblers). The non-clinical sample (i.e., without GD) was recruited from the general population. The questionnaire was diffused on social networks (e.g., WhatsApp, Instagram, e-mail, Facebook, or Linkedln), university bulletin boards, journals for the diffusion of divulgative scientific articles, and websites with divulgation purposes.

The questionnaire was the same for both samples: the group of people with GD (clinical sample) and those without GD (general sample). The sample without GD completed the survey via an online link to the questionnaire or a QR code that accessed the same questionnaire. The GD clinical sample completed the questionnaires both online (*n* = 24) and offline with pen and paper (*n* = 37). According to Herrero (2015), the method of application of the questionnaires (pencil and paper vs. online) does not affect the results obtained. To access the questionnaire, participants had to read the study information and provide informed consent. The duration of the application was about 30 min. The questionnaire included general information about the main goals of the study. We ensured participants' response confidentiality and anonymity and their voluntary participation. No compensation was provided for participation in this study.

### Ethics

The research obtained the ethics committee’s approval from the first author’s university (ref: ETK-17/20–21).

### Data Analysis

First, the correlations between the study variables were analyzed using Pearson's *r*-test. Next, mean differences between the clinical sample and the control sample in gambling motives, violence, and early maladaptive schemas were analyzed using Student's *t*-test. Then, the effect size was calculated using Cohen's *d* (Cohen, 1992), whose parameters establish that values below 0.20 correspond to a small effect size, around 0.50 to a medium effect size, and above 0.80 to a large effect size.

Next, hierarchical regression analyses were performed to test the predictive role of violence and early maladaptive schemas in gambling motives, controlling for the effect of age, employment status, educational level, and marital status. In the first step, age, employment status, educational level, and marital status were introduced to control for their effect; in the second step, violence was added; and in the third step, early maladaptive schemas were added, verifying the changes in *R*^2^ at each step. Three models were analyzed, one for each gambling motive: social motives, enhancement, and coping.

Finally, multiple mediation analyses were performed to analyze the mediating role of early maladaptive schemas, violence, and gambling motives (Preacher & Hayes, 2008). The effect of age, employment status, marital status, and educational level were controlled by introducing them as covariates. First, the relationship between violence (IV) and the mediating variables (early maladaptive schemas) (a-path), and between the mediating variables (early maladaptive schemas) and gambling motives (DV) (b-path) was found to be significant. Then, the total effect between violence (IV) and gambling motives (DV) along with the mediator variables (c-path), and the direct effect between violence (IV) and gambling motives (DV), while controlling for the effect of mediator variables, were found to be significant. When both c-path and c'-path are significant, a partial mediation effect is shown, whereas if c-path is significant and c'-path is not, a total mediation effect is shown. Three models were analyzed, one for each gambling motive (social motives, enhancement, and coping).

## Results

First, the correlation of gambling motives with violence and early maladaptive schemas was analyzed using Pearson's *r* (Table 2). Social gambling motives correlated positively and significantly with violence and the following early maladaptive schemas: enmeshment, subjugation, defectiveness, and dependence. Gambling coping motives correlated positively and significantly with the early maladaptive schema of enmeshment. Gambling enhancement motives correlated positively and significantly with violence and the following early maladaptive schemas: enmeshment, abandonment, abuse, emotional deprivation, subjugation, self-sacrifice, defectiveness, and dependence. All the gambling motives, in turn, correlated positively and significantly with each other.

**Table 2:** Correlation between gambling motives, violence, and early maladaptive schemas

	1	2	3	4	5	6	7	8	9	10	11	12	13	14
1.GM—Social	–													
2.GM-Coping	.50^**^	–												
3.GM-Enhancement	.58^**^	.25^**^	–											
4.Violence	.16^**^	.08	.28^**^	–										
5.EMS-Enmeshment	.13^*^	.22^**^	.17^**^	.11	–									
6.EMS-Abandonment	.10	.05	.13^*^	.16^**^	.41^**^	–								
7.EMS-Abuse	.04	− .09	.14^**^	.21^**^	.23^**^	.57^**^	–							
8.EMS-Emotional Deprivation	.10	− .07	.22^**^	.23^**^	.14^**^	.36^**^	.50^**^	–						
9.EMS-Emotional Inhibition	.03	− .03	.07	.17^**^	.16^**^	.31^**^	.48^**^	.38^**^	–					
10.EMS-Social Isolation	.10	− .04	.11	.20^**^	.23^**^	.39^**^	.56^**^	.57^**^	.48^**^	–				
11.EMS-Subjugation	.13^*^	.01	.28^**^	.27^**^	.33^**^	.55^**^	.59^**^	.54^**^	.39^**^	.64^**^	–			
12.EMS-Self-sacrifice	.09	.01	.23^**^	.05	.27^**^	.44^**^	.38^**^	.32^**^	.16^**^	.28^**^	.43^**^	–		
13.EMS-Failure	− .003	− .01	− .04	.06	.15^**^	.49^**^	.49^**^	.41^**^	.40^**^	.50^**^	.50^**^	.25^**^	–	
14. EMS-Defectiveness	.17^**^	− .02	.25^**^	.19^**^	.21^**^	.37^**^	.51^**^	.49^**^	.51^**^	.6’^**^	.59^**^	.23^**^	.60^**^	–
15.EMS-Dependence	.13^*^	− .07	.15^**^	.15^**^	.24^**^	.49^**^	.42^**^	.26^**^	.33^**^	.43^**^	.51^**^	.25^**^	.54^**^	.59^**^

*Note 1*: GM (Gambling Motives); EMS (Early Maladaptive Schemas)

*Note 2*: **p < .01. *p < .05

Secondly, the mean difference between female gamblers and non-gamblers in gambling motives, violence, and early maladaptive schemas was analyzed using Student's *t*-test (Table 3). The results showed that female gamblers scored significantly higher on gambling motives (social motives, enhancement, and coping), violence, and the following early maladaptive schemas: abuse, emotional deprivation, subjugation, self-sacrifice, and defectiveness. The effect size calculated with Cohen's *d* among significant outcomes was large for enhancement and coping motives and medium for social motives, violence, and all early maladaptive schemas (abuse, emotional deprivation, subjugation, self-sacrifice, and defectiveness schemas).

**Table 3:** Difference in means between gamblers and non-gamblers in gambling motives, violence, and early maladaptive schemas

	Non-gamblers (*n* = 342)	Gamblers (*n* = 61)		
	*M*(*SD*)	*M*(*SD*)	*t(df*)	*d*
1.GM—Social	6.05(1.65)	6.75(2.34)	2.15(1, 65.53)*	− 0.34
2.GM-Coping	5.17(.82)	12.70(4.49)	12.29(1, 53.64)*	− 2.33
3.GM-Enhancement	6.34(2.24)	10.98(4.54)	7.35(1, 57.65)*	− 1.30
4.Violence	16.40(20.86)	37.36(35.78)	3.83(1, 48.90)*	− 0.72
5.EMS-Enmeshment	9.83(4.51)	11.38(6.58)	1.65(1, 60.51)	− 0.27
6.EMS-Abandonment	12.29(6.11)	13.84(7.28)	1.49(1, 68.01)	− 0.23
7.EMS-Abuse	10.70(5.80)	12.37(6.43)	1.93(1, 364)*	− 0.27
8.EMS-Emotional Deprivation	9.79(6.13)	13.70(7.83)	3.50(1, 64.76)*	− 0.55
9.EMS-Emotional Inhibition	11.10(5.80)	11.79(6.67)	0.78(1, 363)	− 0.11
10.EMS-Social Isolation	8.95(4.50)	9.04(4.58)	0.13(1, 364)	− 0.01
11.EMS-Subjugation	9.68(4.99)	12.13(6.40)	2.64(1, 61.78)*	− 0.43
12.EMS-Self-sacrifice	17.51(5.83)	21.37(6.21)	4.37(1, 353)*	− 0.64
13.EMS-Failure	10.29(5.74)	9.10(4.62)	− 1.62(1, 76.05)	0.22
14. EMS-Defectiveness	8.06(3.59)	10.31(5.62)	2.77(1, 56.92)*	− 0.48
15.EMS-Dependence	7.60(3.35)	8.52(4.56)	1.39(1, 60.68)	− 0.23

*Note 1*: GM (Gambling motives); EMS (Early maladaptive schemas)

*Note 2*: *p < .05

Third, hierarchical regression analyses were carried out to test the predictive role of violence and early maladaptive schemas in gambling motives, controlling for the effect of sociodemographic variables (age, employment status, educational level, and marital status) (Tables 4, 5, and 6). In the first step, sociodemographic variables were introduced to control for their effect; in the second step, violence was added; and in the third step, early maladaptive schemas were added. In the case of social motives, the results showed that early maladaptive schemas were predictors of social motives, specifically, enmeshment schema. In the case of enhancement motives, early maladaptive schemas were also predictors of enhancement motives, namely, failure and defectiveness schemas. In the case of coping motives, both violence and early maladaptive schemas were predictors, specifically, subjugation, failure, and defectiveness schemas. As for sociodemographic variables, age had a significant effect in all models, and educational level had a significant effect in the coping motives model.

**Table 4:** Regression of the predictive role of violence and early maladaptive schemes in gambling-coping motives, controlling for the effect of sociodemographic variables

*Coping motives*	*t*	*B*	*SEB*	*Β*	*F(df)*	*R*	*R*^2^	adj. *R*^2^	Change in *R*^2^
*Step 1*					24.03(4, 252)*	0.53	0.28	0.27	0.28*
Age	6.19*	0.09	0.01	0.36					
Study level	− 5.06*	− .86	0.17	− .29					
Employment situation	− .20	− .02	0.12	− .01					
Civil status	.72	− .002	0.003	− .04					
*Step 2*					24.04(5, 251)*	0.57	0.32	0.31	0.05*
Age	5.75*	0.08	0.91	.33					
Study level	− 5.32*	− .88	0.17	− .29					
Employment situation	− .15	− .02	0.12	− .01					
Civil status	− .55	− .002	0.003	− .03					
Violence	4.21*	0.03	0.01	.22					
*Step 3*					10.22(16, 240)*	0.64	0.41	0.37	0.08*
Age	5.49*	0.08	0.02	.33					
Study level	− 5.01*	− .87	.17	− .29					
Employment situation	0.23	0.03	0.12	0.01					
Civil status	− .39	− .001	.003	− .02					
Violence	3.06*	0.02	0.01	0.16					
EMS-Enmeshment	1.60	0.06	0.04	0.09					
EMS-Abandonment	1.37	0.05	0.04	.1’					
EMS-Abuse	− .70	− .03	0.04	− .05					
EMS-Emotional Deprivation	− .91	− .03	0.04	− .06					
EMS-Emotional Inhibition	0.21	0.01	0.04	0.01					
EMS-Social Isolation	− .95	− .06	0.06	− .07					
EMS-Subjugation	2.28*	0.12	0.05	0.17					
EMS-Self-sacrifice	0.32	.’1	0.03	0.02					
EMS-Failure	− 2.61*	− .12	.05	− .18					
EMS-Defectiveness	2.58*	0.18	0.07	0.19					
EMS-Dependence	− .40	− .03	0.07	− .03					

Note 1: EMS (Early Maladaptive Schemas)

Note 2: *p < .05

**Table 5:** Regression of the predictive role of violence and early maladaptive schemas in gambling-enhancement motives, controlling for the effect of sociodemographic variables

*Enhancement motives*	*t*	*B*	*SEB*	*Β*	*F(df)*	*R*	*R*^2^	adj. *R*^2^	Change in *R*^2^
*Step 1*					4.45(4, 251)*	.26	.07	.05	.06*
Age	2.63*	0.04	0.02	0.18					
Study level	− 1.62	− .32	.20	− .11					
Employment situation	− 1.09	− .15	.14	− .07					
Civil status	− .66	− .02	.003	− .04					
*Step 2*					4.21(5, 250)*	0.28	0.08	0.06	0.01
Age	2.36*	0.04	0.02	0.16					
Study level	− 1.74	− .34	.20	− .11					
Employment situation	− 1.05	− .15	.14	− .07					
Civil status	− .59	− .02	.003	− .04					
Violence	1.76	0.02	0.01	0.11					
*Step 3*					2.40(16, 239)*	0.37	0.14	0.08	0.06
Age	2.44*	0.04	0.02	0.17					
Study level	− 1.70	− .36	.21	− .12					
Employment situation	− .72	− .10	.14	− .05					
Civil status	− .75	− .002	.003	− .05					
Violence	1.17	0.01	0.01	0.08					
EMS-Enmeshment	− .07	− .003	0.05	− .01					
EMS-Abandonment	1,77	0.08	0.05	0.16					
EMS-Abuse	− 1.16	− .06	0.05	− .10					
EMS-Emotional Deprivation	− .74	0.,03	0.04	− .06					
EMS-Emotional Inhibition	− 1.08	0.,05	0.04	− .08					
EMS-Social Isolation	0.83	0,06	0.07	0.07					
EMS-Subjugation	0.34	0.02	0.06	0.03					
EMS-Self-sacrifice	− .39	− .02	0.04	− .03					
EMS-Failure	− 2.29*	− .13	0.06	− .19					
EMS-Defectiveness	2.03*	0.16	0.08	0.18					
EMS-Dependence	1.11	0.09	0.08	0.09					

Note 1: EMS (Early Maladaptive Schemas)

Note 2: *p < .05

**Table 6:** Regression of the predictive role of violence and early maladaptive schemas in gambling-social motives, controlling the effect of sociodemographic variable

*Social motives*	*t*	*B*	*SEB*	*Β*	*F(df)*	*R*	*R*^2^	adj. *R*^2^	Change in *R*^2^
*Step 1*					2.68(4, 253)*	.20	.04	.03	.04*
Age	2.88*	0.03	0.01	0.19					
Study level	2.26*	0.25	0.11	0.15					
Employment situation	1.07	0.09	0.08	0.07					
Civil status	− .44	− .001	.002	− .03					
*Step 2*					2.18(5, 252)	0.20	0.04	0.02	0.001
Age	2.78*	0.03	0.01	0.19					
Study level	2.24*	0.25	0.11	0.15					
Employment situation	1.08	0.09	0.08	0.07					
Civil status	− .42	− .001	0.002	− .03					
Violence	.45	.002	0.005	0.03					
*Step 3*					1.82(16, 241)*	0.33	0.11	0.05	0.07
Age	2.56*	0.03	0.01	0.18					
Study level	1.14	0.14	0.12	0.08					
Employment situation	0.93	0.08	0.08	0.06					
Civil status	− .32	− .001	.002	− .02					
Violence	0.72	0.004	0.01	0.05					
EMS-Enmeshment	2.68*	0.07	0.03	0.19					
EMS-Abandonment	1.23	0.03	0.03	0.11					
EMS-Abuse	− 1.81	−.05	0.03	− .15					
EMS-Emotional Deprivation	− 1.17	− .03	.03	− .09					
EMS-Emotional Inhibition	0.36	0.01	0.02	0.03					
EMS-Social Isolation	− .68	− .03	0.04	− .06					
EMS-Subjugation	0.51	0.02	0.04	0.05					
EMS-Self-sacrifice	− .54	− .01	0.02	− .04					
EMS-Failure	0.89	0.03	0.03	0.07					
EMS-Defectiveness	0.51	0.02	0.05	0.05					
EMS-Dependence	1.64	− .08	0.05	− .13					

Note 1: EMS (Early Maladaptive Schemas)

Note 2: *p < .05

**Fig. 1:**
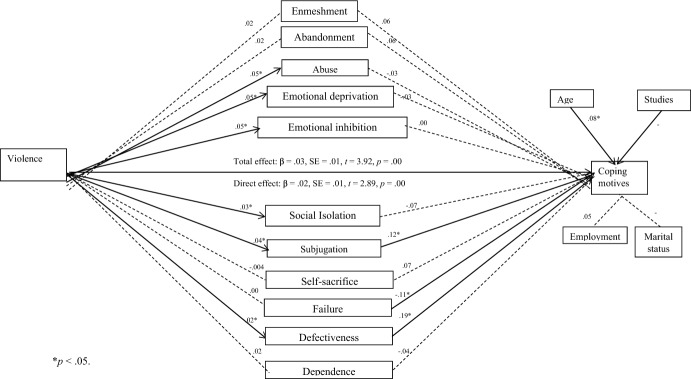
Mediational effect of early maladaptive schemas between violence and coping motives controlling for sociodemographic variables

Fourth, multiple mediation analyses were conducted to test the mediating role of early maladaptive schemas between violence and gambling motives, controlling for the effect of sociodemographic variables (Fig. 1). First, the relationship between violence and early maladaptive schemas (path-a), and between early maladaptive schemas and gambling motives (path-b) was verified. Next, the relationship between violence and gambling motives (path-c), and between violence and gambling motives when controlling for the effect of emotional dependence (path-c') was verified. The results showed that early maladaptive schemas partially mediated between violence and coping motives, with subjugation and defectiveness standing out as significant mediators of the relationship. Age and educational level were, in turn, significant predictors of coping motives.

## Discussion

The first objective of the study focused on analyzing the correlations between the study variables. The data reported in the present study indicated positive relationships between social gambling motives and received psychological violence and with the early maladaptive schemas of enmeshment, subjugation, defectiveness, and dependence. Likewise, gambling coping motives were positively related to early maladaptive attachment schemas. Finally, gambling enhancement motives correlated positively with received psychological violence and the early maladaptive schemas of enmeshment, abandonment, abuse, emotional deprivation, subjugation, self-sacrifice, defectiveness, and dependence.

Studies of early maladaptive schemas to gambling motives in women represent a novel aspect of the present study. However, these results could be explained by previous studies that have linked early maladaptive schemas and various addictive behaviors, such as gambling addiction (Viera et al., 2023). The results obtained coincide with a previous study finding that all the schemas belonging to the domains of disconnection and rejection and autonomy impairment positively and significantly correlated with behavioral addictions (Estévez & Calvete, 2007; Ostovar et al., 2021; Sakulsriprasert et al., 2023). The present study also found a predominance of schemas belonging to the disconnection/rejection and impaired autonomy dimensions in women’s gambling motives.

Of note is the presence of the enmeshment schema in all three gambling motives. Enmeshment schema refers to excessive emotional involvement and closeness with one or more significant others at the expense of one's own individualization or development. This could be related to the fact that 70% of people with problematic gambling have been found to present a predominantly anxious-ambivalent insecure attachment (Di Trani et al., 2017). This insecure attachment style is related to enmeshment schemas, dependence on others, and impulsive behaviors (Estevez et al., 2019. Likewise, previous studies have found that insecure attachment predicts increased gambling coping motives and greater severity of gambling (Keough et al., 2018; Marchica et al., 2020).

The increase in social and coping motives as the received psychological violence increases could be explained by the fact that gamblers may engage in gambling to obtain positive emotions (Rocha et al., 2023). In addition, violence is linked to more social isolation, so these women may use gambling to socialize (Eckhardt et al., 2022). Along these lines, it has been noted that loneliness and isolation play a crucial role in women's gambling experiences. That is, women are motivated to gamble to escape from loneliness and feel socially accepted. In addition, they maintain their gambling behavior out of fear of feeling lonely. This fear of loneliness has been found to increase with age (Kim et al., 2016). Thus, the results show the centrality of relational aspects in both the origin and maintenance of gambling among women who suffer gender-based violence (Odoardi & Albasi, 2013).

The study's second objective was to analyze the mean differences in gambling motives, received psychological violence, and early maladaptive schemas between women in the clinical group and the control group. Our findings indicate that female gamblers obtain higher scores on gambling motives—especially coping and enhancement—, received violence, and early maladaptive schemas of abuse, emotional deprivation, subjugation, self-sacrifice, and defectiveness. These results are consistent with previous studies in which women with problem gambling presented higher levels of gender-based violence victimization (O'Mullan et al., 2022) and higher scores on all gambling motives (Macía et al., 2023). Likewise, the results obtained in this study align with previous studies indicating that non-addicted women’s main motivation for engaging in gambling behaviors was money, followed by feelings and socialization. On the other hand, women addicted to gambling were mainly motivated by feelings, escape, and socialization (Leonek-Kuleta, 2022). It should be noted that gambling motives varied according to the women’s age. Gambling for positive feelings and socialization are more frequent motives among younger female gamblers, whereas support for noble causes and escape motives were more frequent among older women and housewives (Hagfors et al., 2022). Likewise, it has been found that middle-aged female gamblers who were motivated by winning money or entertainment, in general, had a high risk for GD (Hing et al., 2016). Higher scores on early maladaptive schemas of abuse, emotional deprivation, subjugation, self-sacrifice, and defectiveness in women with GD represent a novel aspect of the present study. These results could be due to the fact that women with gambling problems report greater childhood traumatic experiences than women without gambling problems (Hodgins et al., 2010). These traumatic experiences could instill dysfunctional beliefs about the self and others (Estevez et al., 2019). In turn, such traumas are closely linked to gambling severity (Imperatori et al., 2017).

The third objective of the present study is to analyze the predictive role of received psychological violence and schemas in gambling motives. In the case of social motives, the results showed that early maladaptive schemas—specifically, enmeshment schemas—predicted social motives. In the case of enhancement motives, early maladaptive schemas—namely, failure and defectiveness—were also predictors. In the case of coping motives, both violence and early maladaptive schemas— namely, subjugation, failure, and defectiveness schemas—were predictors. These results are consistent with previous studies in which early maladaptive schemas predicted women’s gambling motives (Estevez et al., 2023). On the one hand, schemas of failure and defectiveness learned at early ages could predict women's engagement in gambling behaviors as a way to experience positive feelings (Rocha et al., 2023). On the other hand, received psychological partner violence and schemas of subjugation, failure, and defectiveness could also predict women's engagement in gambling as a way to cope with painful and negative emotions produced by experiences of violence and dysfunctional beliefs about oneself (Rapinda et al., 2023). In addition, gender-based violence has been found to motivate women's gambling as a way to physically and psychologically escape from it and regain control over their lives. However, gambling in women has been found to exacerbate gender-based violence (Hing et al., 2023). Finally, the enmeshment schema could predict women's engagement in gambling for social reasons due to the core belief underlying this schema that refers to the impossibility of being happy without the continuous and extreme support and proximity of a significant other (Young et al., 2013). It is important to highlight that each gambling motive was predicted by a specific early maladaptive schema and that only received psychological violence significantly predicted coping motives. These results suggest that women who have suffered psychological violence by their partner could have a specific gambling profile.

Finally, the fourth objective of the study was to analyze the mediating role of early maladaptive schemas in the relationship between violence and gambling motives. The results showed that early maladaptive schemas partially mediated between violence and coping motives, highlighting subjugation and defectiveness as significant mediators in this relationship. Previous studies have indicated that women gamble in response to intimate partner violence (Hing et al., 2023). These results could be explained because gender violence can establish dysfunctional beliefs about being imperfect, not valuable or loved, and inferior in important life aspects and the need to renounce one's rights and give in to coercion or others’ control to avoid negative consequences (Momeñe et al., 2021). These beliefs can generate high negative affect and low self-esteem in women, leading to motivational orientations that predict engagement in gambling to cope with negative affect (Rapinda et al., 2023; Rodríguez et al., 2015).

### Limitations

The present study is not without limitations. Firstly, the nature of the study is cross-sectional, which hinders obtaining causal relationships between the study variables. Future studies are recommended to replicate the present study using longitudinal methodology. A second limitation is related to the collection of information through self-reports. Social desirability may have influenced the responses to the questionnaires. In addition, it was decided to use a previous version of the GMQ questionnaire. Third, there were more women in the control group than in the clinical group. It is recommended that future studies replicate the research equating the number of participants in both groups. Finally, we note that women who suffer gender violence may be reluctant to verbalize the abuse (Hing et al., 2022). Furthermore, women show greater shame and guilt-proneness about their gambling behavior than men, which may influence its concealment (Kushnir et al., 2016).

## Conclusions

In conclusion, this study opens new research perspectives to better understand the mechanisms underlying the gambling motives of women who suffer gender violence and has clinical implications regarding prevention and treatment. A cognitive intervention focused on beliefs related to being imperfect, not valuable or loved, and that they must give in to the control and coercion of others to avoid negative consequences could be beneficial for women suffering gender violence and who have engaged in gambling to deal with negative emotions. These results are novel and useful due to the few studies that use a clinical sample of women with a gambling problem.

## Data Availability

All data generated or analyzed during the present study are available on request from the reviewers.
